# Thermally Activable Bistetrazoles for Elastomers Crosslinking

**DOI:** 10.3390/polym14142919

**Published:** 2022-07-19

**Authors:** Mauro Monti, Luca Giannini, Luciano Tadiello, Silvia Guerra, Antonio Papagni, Luca Vaghi

**Affiliations:** 1 Department of Materials Science, University of Milano-Bicocca, Via Cozzi 55, 20125 Milano, Italy; mauro.monti@unimib.it (M.M.); antonio.papagni@unimib.it (A.P.); 2 Pirelli Tyre S.p.A., Viale Sarca 222, 20126 Milano, Italy; luca.giannini@pirelli.com (L.G.); luciano.tadiello@pirelli.com (L.T.); silvia.guerra@pirelli.com (S.G.)

**Keywords:** elastomers crosslinking, styrene-butadiene rubber, tetrazoles, nitrilimines, cycloaddition, curing, mechanical properties

## Abstract

Sulfur vulcanization is the most used method for curing of natural and synthetic rubbers. The crosslinking degree achieved is usually controlled by adding proper quantities of accelerants, activators, co-activators, retardants, and inhibitors, and influences the hardness, elasticity, hysteresis of elastomers and, consequently, the properties and behavior of the materials that incorporate them. Despite the fine tuning pursued over the years, sulfur crosslinking is still difficult to control both in terms of degree and homogeneity of cross-link. Addition of thermally activable bifunctional reagents able to crosslink the polymer matrix through covalent bonds could be a strategy to modulate and control finely the reticulation grade of elastomers. Tetrazoles can form highly reactive nitrilimines by thermal treatment at appropriate temperature, which can react with the vinyl double bonds present in the rubber. In this work a set of bis-tetrazoles were synthesized and those with the right activation temperatures were used for the curing of styrene-butadiene rubber, acting both as single crosslinkers and together with classic sulfur-based ones. The addition of bistetrazoles simplified and made more efficient the compounding process, allowing to prolong the mixing until optimum dispersion and homogeneity were obtained. Moreover, they led to an improvement in the hysteretic properties of the compound and to the reduction of the non-linearity of the dynamic behavior (Payne effect).

## 1. Introduction

In the tire industry, sulfur crosslinking is a process commonly used to control the mechanical properties of compounds [1]. Cross-linking influences the hardness, elasticity, hysteresis of elastomeric materials and, consequently, the properties and behavior of the tire that incorporates them [2]. Over the years, several additives were proposed to improve the crosslinking process such as activators, accelerators, and retarding agents [3,4,5,6]. Even with the use of additives, conventional crosslinking systems (based on sulfur) are however not completely satisfactory since they are not very versatile and difficult to control both in terms of degree and homogeneity of cross-link and in activation temperatures [7,8,9]. These latter aspects introduce problems of scorching due to early crosslinking and not optimal dispersion of sulfur ending up in materials with poor hysteretic properties. Typically, the cross-linking agent (sulfur) and the cross-linking additives are incorporated into the mix in one or more stages of the production process at controlled temperature, generally not exceeding 110 °C, and for limited mixing times to avoid triggering the crosslinking reactions early [10]. However, the low solubility of sulfur in elastomeric compounds together with the mild mixing conditions make not always optimal the sulfur dispersion. For example, in the case of mixtures of elastomers with different polarity, undesired accumulation of sulfur can occur in the more affine elastomeric phase [11,12]. Because of an unsatisfactory distribution of sulfur, the cross-linked final material may not have the desired properties, in particular it can be characterized by a marked hysteresis, i.e., showing an increase in the heat dissipated under dynamic conditions. Furthermore, the ultimate properties such as breaking load and elongation at break can be compromised by a non-homogeneous distribution of the vulcanizers. Finally, the migration of some components to the interface between different compounds can cause problems of stiffening at the interfaces reducing their mechanical strength. The problem appears where there is contact between compounds rich in accelerators but poor in sulfur and compounds rich in sulfur and poor in accelerators (example tread/substrate), in tires this translates into high rolling resistance, and overall, greater fuel consumption [13]. Currently, most vehicle manufacturers increasingly require their suppliers to develop low rolling resistance tires to reduce fuel consumption and environmental impact. A possible approach to decrease the hysteresis of elastomeric materials is based on the use of special reinforcing fillers that can partially or completely replace standard fillers such as carbon black and silica and give less hysteresis while maintaining enough reinforcement. However, the need remains to further reduce the rolling resistance of the tires and therefore to produce increasingly eco-compatible tires and to increase the useful life of the tire by maximizing the breaking resistance of all its parts.

**Scheme 1:**
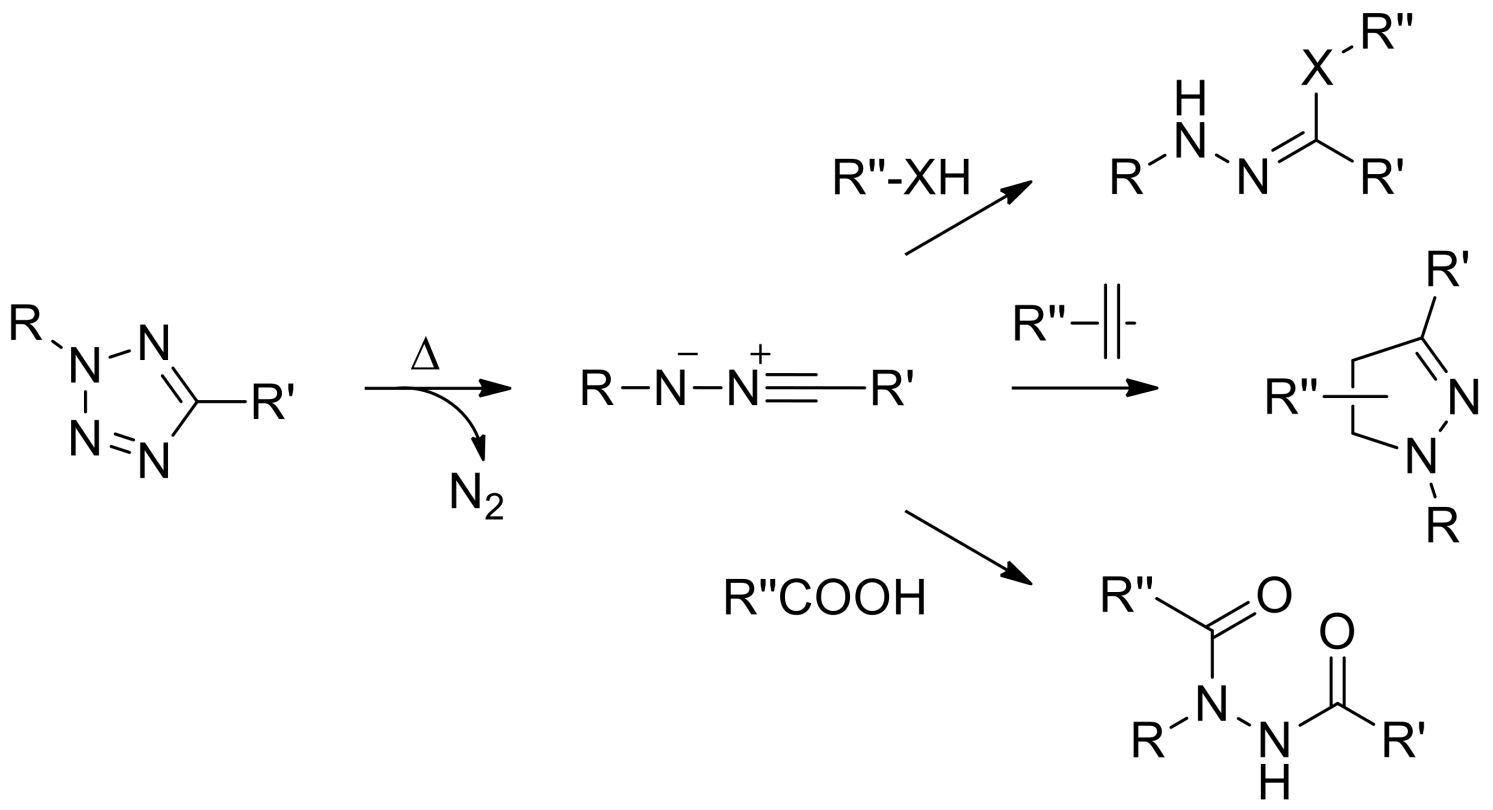
Thermally induced nitrilimine generation and reactivity towards functional groups.

Addition of molecular species able to covalently cross-link the elastomers at the right stage of the process could be the solution of the aforementioned problems. We were attracted by tetrazole moiety, that can be thermally activated to form a nitrilimine intermediate displaying high reactivity toward several functional groups (Scheme 1) [14,15,16,17,18], including 3+2 cycloaddition to pyrazoline in presence of C-C double bonds, which are abundant in polymers used for tires. Moreover, with the same type of substituents, the less the double bond is substituted, the better the reactivity of the cycloaddition [19,20,21,22], assuming a selectivity toward the vinyl bonds of elastomers.

Few studies reported the use of tetrazoles with elastomeric materials. Stille et al. showed the preparation of synthetic elastomers which incorporate tetrazole pendants in the polymeric chains [23]. Copolymerization of tetrazoles substituted styrene with styrene, vinylidene chloride, and acrylonitrile gave functionalized copolymers, their heating at around 200 °C led to materials with properties comparable to conventional SBR (styrene-butadiene rubber) elastomers treated with sulfur and zinc oxide. The prepared materials did not require crosslinking additives, and nothing was reported about possible interaction of the materials with fillers and other materials typically present in a technical compound. More recently was reported the synthesis of high molecular weight NBR (nitrile butadiene rubber) by coupling short NBR chains terminated with a 2,5-disubstituted tetrazole on a maleimide linker [24]. Photochemical activation gave nitrilimine which reacted selectively with the double bonds of the linker instead of the much more numerous ones of the NBR. No mechanical characterization or technical applications of the prepared materials were discussed.

**Figure 1:**
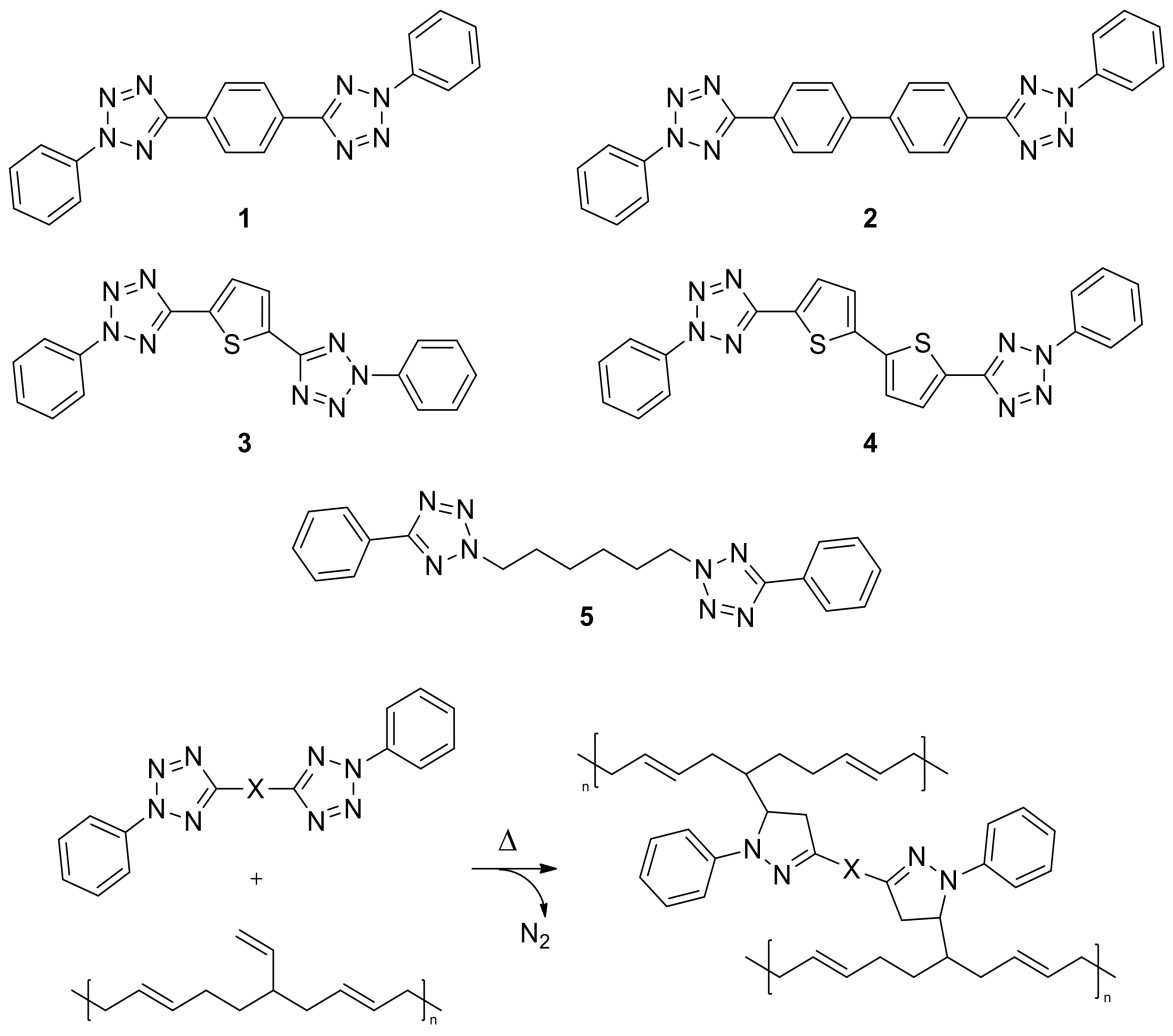
Structures of bistetrazoles **1–5** used in this study and expected reactivity toward polymeric chains.

We hypothesized that linear organic systems containing two tetrazole moieties at the ends, activable at the right temperature, could be ideal crosslinking agents for tire elastomers. We report herein the preparation of bis-tetrazoles **1–5** (Figure 1), the study of their activation temperatures, and their application in the curing of styrene-butadiene rubber under different operating conditions.

## 2. Materials and Methods

### 2.1. General

All reagents and solvents for synthesis were purchased from commercial sources (Merck Life Science S.r.l., Milan, Italy; Fluorochem Ltd., Hadfield, UK; and TCI Europe N.V., Zwijndrecht, Belgium) and used without further purification. Chromatographic purifications were performed using Merck 9385 silica gel, pore size 60 Å (230–400 mesh) (Merck Life Science S.r.l., Milan, Italy). Melting points were measured with a Stanford Research Systems Optimelt apparatus (SRS, Sunnyvale, CA, USA). IR spectra were recorded with a PerkinElmer Spectrum 100 FT-IR spectrometer equipped with universal ATR sampling accessory (PerkinElmer Inc., Waltham, MA, USA). ^1^H and ^13^C spectra were recorded with a Bruker AVANCE III HD 400 MHz spectrometer (^1^H: 400 MHz, ^13^C: 101 MHz) (Bruker corp., Billerica, MA, USA). Elemental analyses were obtained with an Elementar vario MICRO cube instrument (Elementar Analysensysteme GmbH, Langenselbold, Germany). TGA analyses were performed using Mettler Toledo TGA/DSC1 StarE (Mettler-Toledo International Inc., Greifensee, Switzerland). The following ingredients were used in the preparation of elastomeric compounds: SSBR (extended solution styrene-butadiene copolymer with 37.5 phr of TDAE oil for every 100 phr of Dow SE SLR-4630 dry elastomeric polymer, Trinseo PLC, Berwyn, PA, USA); silica (Zeosil 1165 MP, Rhodia Operations S.A.S., Aubervilliers, France); stearic acid (Temix Oleo SRL, Milano, Italy); 6PPD (Eastman, Kingsport, TN, US); ZnO (80% zinc oxide, 20% polymeric binder and dispersing agent, Lanxess AG, Cologne, Germany); TESPD (Jingzhou Jianghan Fine Chemical Co., Ltd., Jingzhou, China); CBS (Dulso, a.s., Šaľa, SVK); sulfur (Crystex OT33, Eastman, Kingsport, TN, USA). The mixing was carried out using an internal Brabender laboratory tangential rotor mixer (60 mL mixing chamber) (Brabender GmbH & Co. KG, Duisburg, Germany). The dynamic mechanical properties were evaluated using a RPA 2000 rheometer (Alpha Technologies Services LLC, Hudson, OH, USA).

### 2.2. Synthesis of Cross-Linkers 1–5

The synthetic procedures, complete characterizations, thermo gravimetric analyses and ^1^H NMR, ^13^C NMR, and ATR-FTIR spectra of **1**, **2**, **3**, **4**, and **5** are reported in the Supplementary Materials.

### 2.3. Mixing Procedure

The mixing was carried out in several stages using an internal laboratory mixer with Brabender tangential rotors (60 mL mixing chamber). In the first mixing phase, 50% of the elastomer was introduced and chewed for 30 s at 140 °C (set temperature). In the following mixing phase, bistetrazole, silica, silane, and the remaining elastomer were added. The mixing was continued for 2 min, at 140 °C. Subsequently, the antioxidant, ZnO, and stearic acid were introduced. The mixing was continued for about 2 min, until the reaction between silica and silane was completed, at 140 °C after which the compounds, called first phase compounds, were discharged and tested for their dynamic properties. After 12–24 h, the vulcanizer (sulfur) and accelerator were introduced into the first phase compounds, and the mixing continued for about 3 min, obtaining the final compounds at 90 °C. Then final compounds were re-tested for their dynamic properties.

### 2.4. Dynamic Mechanical Properties

#### 2.4.1. Single Temperature Curing

The first stage and the final elastomeric compounds were tested for the dynamic mechanical rheological properties according to the following conditions: (1) Deformation cycle up to 100% at 70 °C, 10 Hz to determine the rheological properties of raw compounds; (2) heating at 190 °C for standard SBR and at Ta when **1–4** are present, for 30 min to obtain the cross-linking; (3) deformation cycle up to 10% at 70 °C, 10 Hz to determine the dynamic properties after the cross-linking.

#### 2.4.2. Separate Temperature Curing

The dynamic mechanical properties at separate temperature were detected according to the following conditions: (1) Deformation cycle up to 100% at 70 °C, 10 Hz to determine the rheological properties of raw compounds; (2) heating at 150 °C for 40 min to obtain a first low T cross-linking in which only the sulfur vulcanization system is activated; (3) deformation cycle up to 10% at 70 °C, 10 Hz to determine the dynamic properties after the first cross-linking; (4) heating at 190 °C for 30 min to achieve the cross-linking by the bis-tetrazole cross-linker; (5) deformation cycle up to 10% at 70 °C, 10 Hz to determine the dynamic properties after the second cross-linking stage.

## 3. Results and Discussion

### 3.1. Synthesis

**Scheme 2:**
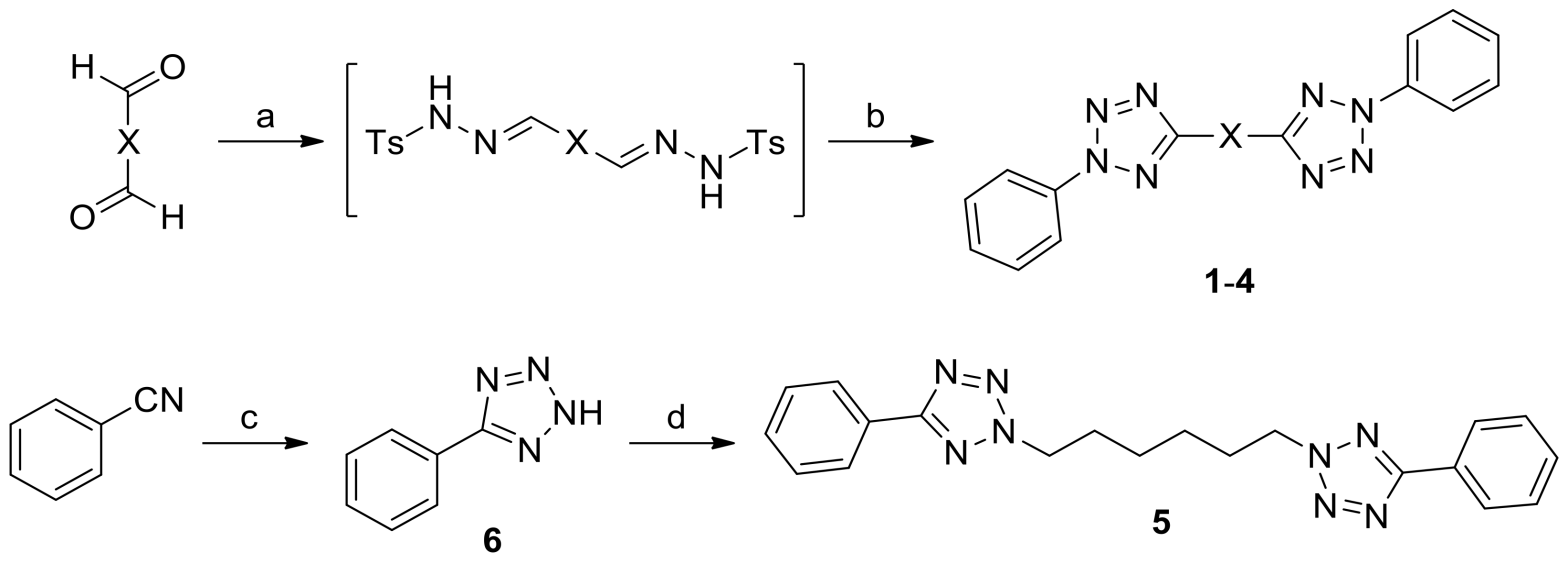
Synthesis of bistetrazoles **1–5**. Reagents and conditions: (**a**) *p*-toluenesulfonyl hydrazide, EtOH, reflux, 5 h; (**b**) benzenediazonium chloride, pyridine, 0 °C to rt, overnight; (**c**) ZnBr_2_, NaN_3_, H_2_O, reflux, 48 h; (**d**) 1,6-dibromohexane, K_2_CO_3_, DMF, rt, 24 h.

Bistetrazoles **1–4** were synthesized by treatment of dialdehydebistosylhydrazone with diazonium aniline salt, starting from the appropriate dialdehyde (Scheme 2), i.e., terephthalaldehyde, biphenyl-4,4’-dicarbaldehyde, thiophene-2,5-dicarbaldehyde, and 2,2’-bithiophene-5,5’-dicarbaldehyde for **1**, **2**, **3,** and **4**, respectively. The preparation of **5** involves first the synthesis of phenyltetrazole (**6**) by treatment of benzonitrile with sodium azide in presence of zinc bromide [25], then alkylation with 1,6-dibromohexane (Scheme 2).

### 3.2. Thermal Activation

Activation temperature (*T*_a_) of bistetrazoles is a key factor for effectiveness crosslinking. It must be not lower than 100 °C, since it could give rise to crosslinking reactions too early, during the mixing phases of the components prior to vulcanization. Early crosslinking would make the compound difficult to work with, both in the unloading phases from the internal mixer and in the extrusion processes of the semi-finished products, thus compromising the integrity of the finished tire, and not higher than 210 °C, to avoid degradation of the elastomers.

Depending on the specific application, the cross-linking agents may have an ideal activation temperature between 100 °C and 130 °C, to allow partial pre-crosslink of the compound, increasing its viscosity in a controlled way before conventional vulcanization. In this case, the mixing phases will be carried out at a controlled temperature, near that of activation. In particular, such a low activation temperature causes partial pre-crosslinking of the thinner semi-finished products, improving their handling without compromising their adhesiveness and ability to co-vulcanize with the other layers of the tire and with the reinforcement elements. An activation temperature similar to a classic curing system (between 130 °C and 170 °C) is used, to allow the mixture to be crosslinked with both the cross-linking systems (conventional sulfur-based and bistetrazoles) in a single step, resulting in a more uniform and increased cross-linking with respect to classical methods. An activation temperature higher than typical sulfur cure (from 170 °C to 210 °C) is used to allow to proceed with the conventional preparation steps without having to control the temperature strictly, if not to avoid the early sulfur vulcanization of the mixture (mixing T preferably below 120 °C). Such a high activation temperature allows to prepare compounds already vulcanized with sulfur but still capable of cross-linking when, for example, subjected to particularly stressful conditions of use, with overheating beyond that specific activation temperature. In this way it could be possible to remedy the degradation of the material under stress owing to the formation of new bonds during the use of the tire.

**Figure 2:**
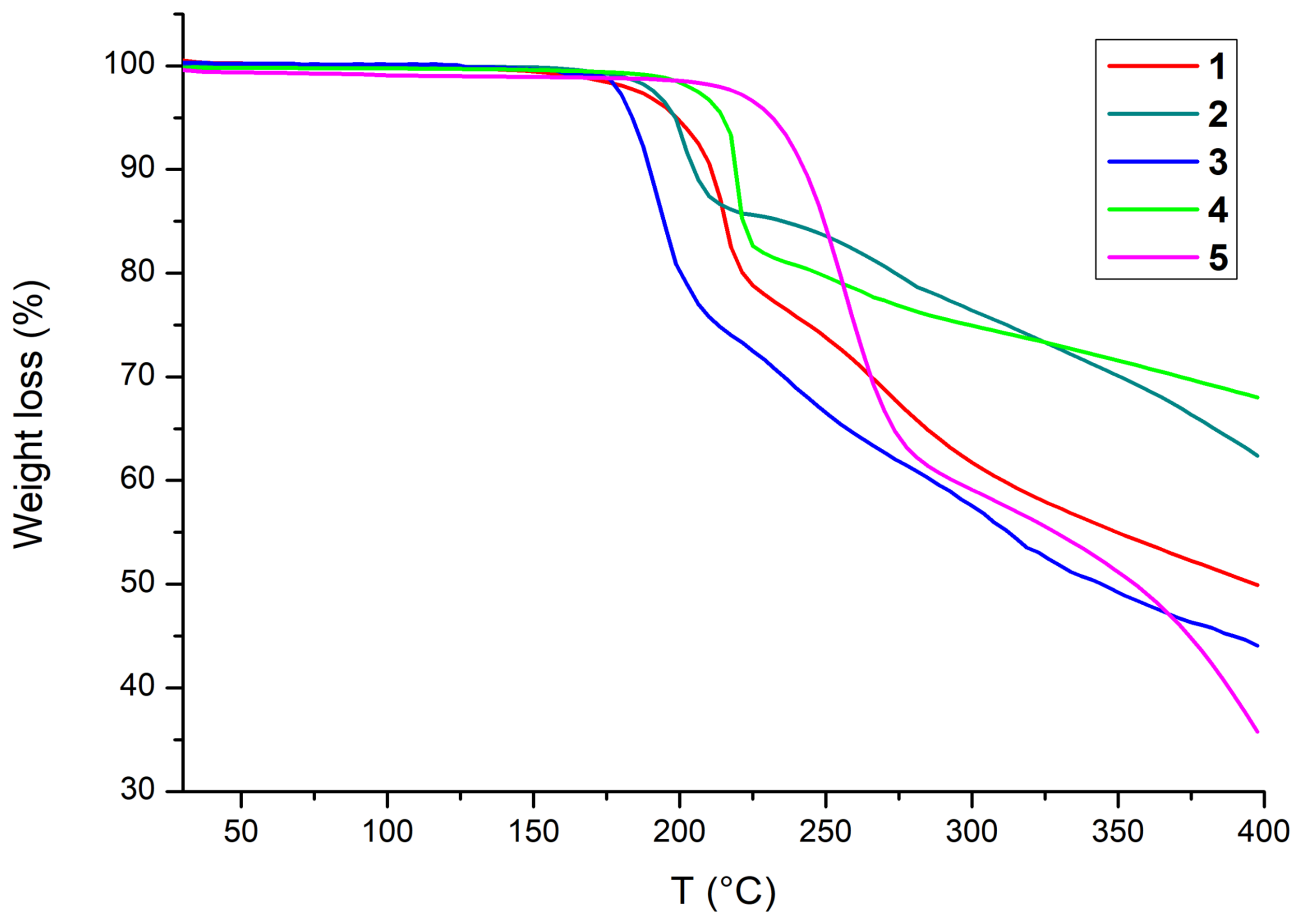
TGA analysis of compounds **1–5** (30–400 °C, 5 °C/min; N_2_).

Thermogravimetric analysis (TGA) was used to detect the activation temperatures of bistetrazoles **1–5** (Figure 2 and Table 1), the start of weight loss indicates the formation of nitrilimines through release of N_2_ molecules.

Bistetrazoles **1** and **2** revealed a T_a_ of 185 °C, while activation of **3** and **4** occurs at 170 °C and 190 °C, respectively. T_a_ should permit both the covulcanization with sulfur and post vulcanization crosslinking. Meanwhile the detected T_a_ of **5** (230 °C) is too high for any curing application and was not further investigated. Such a high T_a_ could be caused by the aliphatic chain instead of aromatic connecting the two tetrazole moieties.

### 3.3. SBR Curing with Bis-Tetrazoles

To test the efficacy of crosslinking by **1–4,** first stage elastomeric compounds were prepared, i.e., without sulfur and additives needed for sulfur vulcanization. The quantities of the various components expressed in parts per hundred rubber (phr) are reported in Table 2.

**Figure 3:**
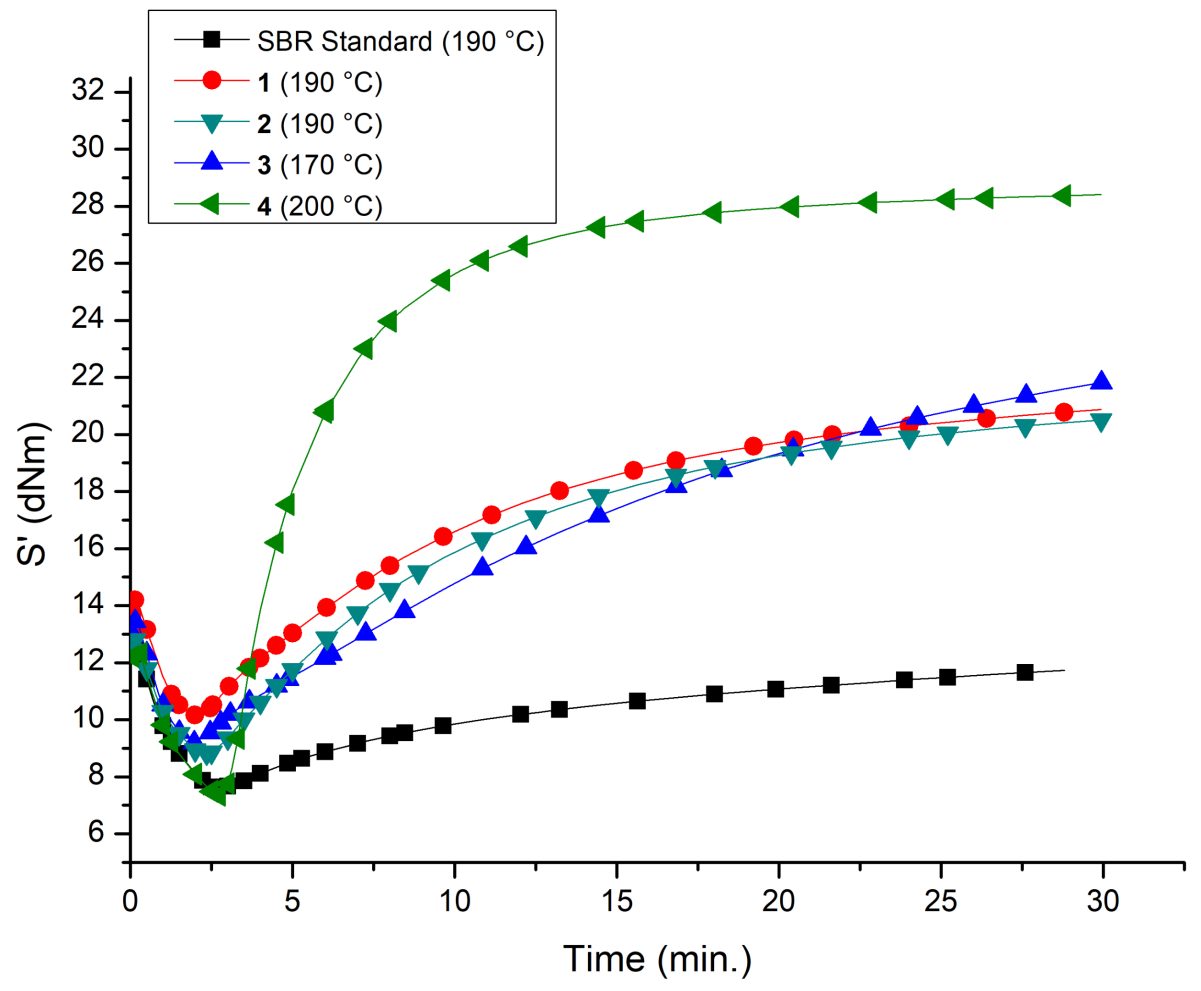
Torque crosslinking curves of first stage compounds.

The prepared first stage elastomeric compounds were then subjected to curing for testing the rheological properties at 190 °C for reference SBR, and at T_a_ for compounds containing **1–4**. The torque crosslinking curves (S’) are reported in Figure 3. 

It is evident from the traces in Figure 3 that with respect to standard SBR, first stage compounds containing **1–4** display a higher degree of crosslinking, with increasing torque over the time. The steepest growth observed in the case of **4** is almost certainly due to the faster kinetic induced by the higher temperature.

Next, final compounds with complete ingredients, i.e., sulfur and all additives normally used in curing of SBR, were prepared (Table 3). 

**Figure 4:**
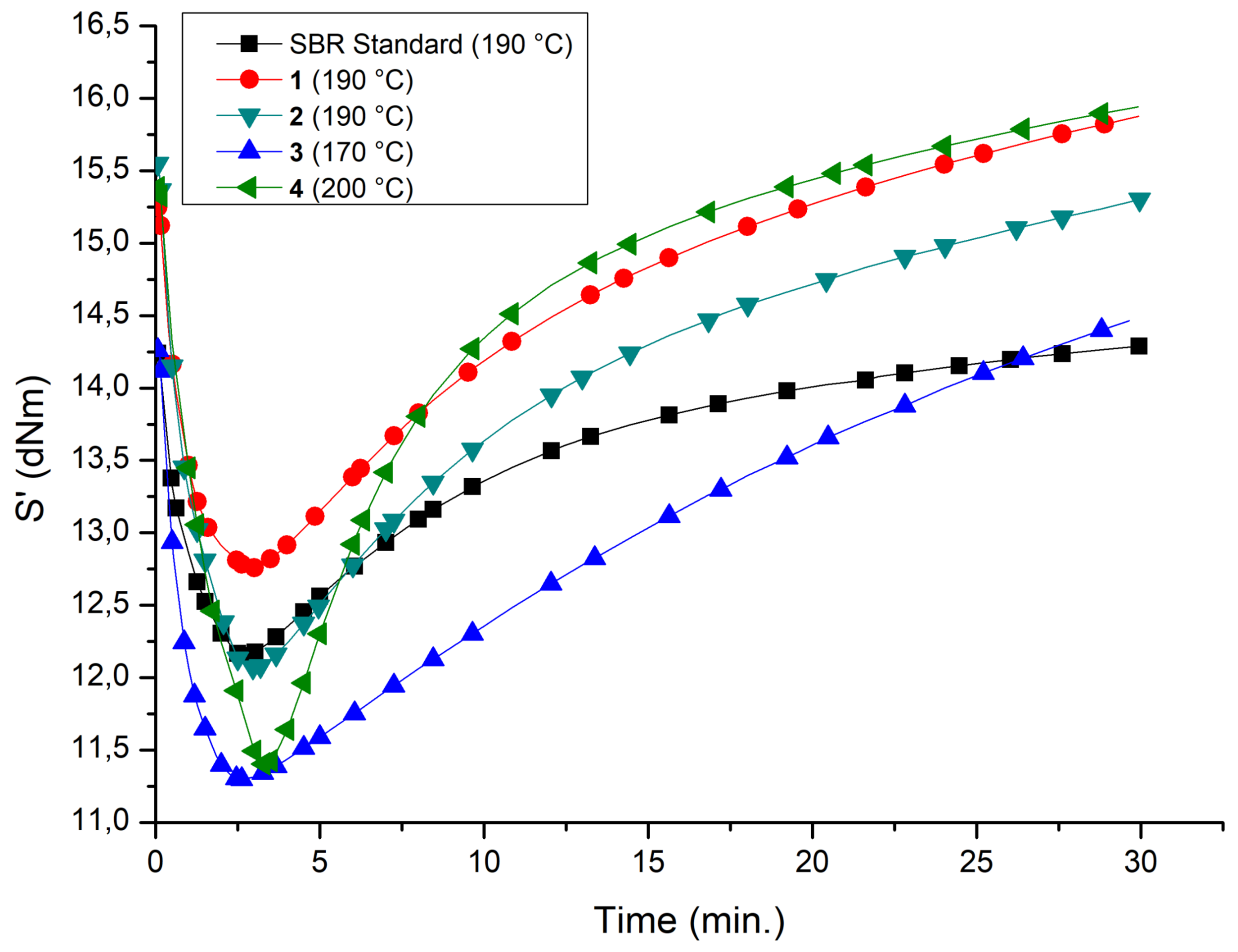
Torque crosslinking curves of final compounds.

The prepared final elastomeric compounds were in turn cured to measure the torque crosslinking curve (S’, Figure 4) in the same conditions as used for first stage elastomeric compounds, to evaluate the possible cross linking synergic effect of **1–4** in the presence of all vulcanization ingredients. 

Moreover, in this case an evident effect can be seen from S’ curve with respect to standard cured SBR. While curves of elastomeric compounds treated with **3** and **4** are affected by different curing temperatures, direct comparison can be especially done between standard SBR and mixtures with **1** and **2**, treated at the same temperature. The extra crosslinking affected by bistetrazoles results in greater growth and higher final values of S’ curve.

### 3.4. In Depth Dynamic Mechanical Properties of Elastomeric Compunds Containing 1

#### 3.4.1. Single Temperature Curing

**Figure 5:**
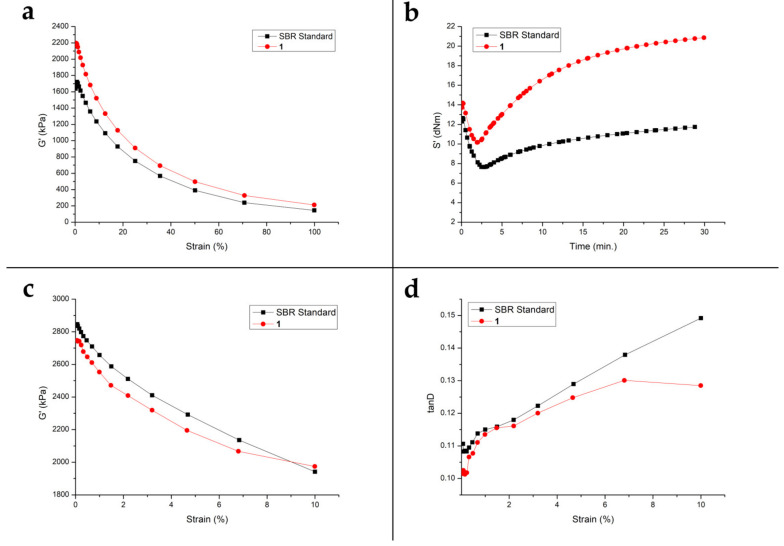
Dynamic mechanical properties of first stage compound treated with bistetrazole **1** compared to first stage standard SBR. (**a**) G’ measured before curing; (**b**) S’ at 190 °C; (**c**) G’ measured after curing at 190 °C; (**d**) tan delta after curing at 190 °C.

**Figure 6:**
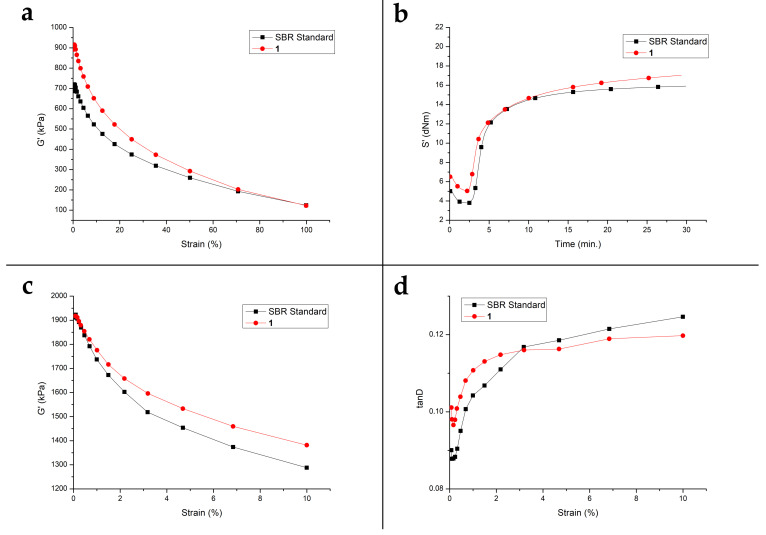
Dynamic mechanical properties of final compound treated with bistetrazole **1** compared to final standard SBR. (**a**) G’ measured before curing; (**b**) S’ at 190 °C; (**c**) G’ measured after curing at 190 °C; (**d**) tan delta after curing at 190 °C.

The first stage and final elastomeric compounds prepared with **1** were compared thoroughly to standard SBR for dynamic mechanical properties (Figure 5 and Figure 6).

In both first stage compounds and final compounds, the addition of the bis-tetrazole crosslinking agent **1** leads to an elastic modulus at low deformations higher than that of the respective reference SBR (Figure 5a and Figure 6a). The difference in elastic modulus in these raw compounds tends to decrease with increasing deformation, so the addition of **1** tends to increase the Payne effect. This effect could be due to the low solubility of **1** in the mixture at 70 °C, behaving like a filler. However, the increased Payne effect of the raw compounds does not have negative consequences and can indeed be useful in some semi-finished products, translating into a reduction of the risk of deformation of the semi-finished products during handling and storage.

The crosslinking curves measured at 190 °C (Figure 5b and Figure 6b) show the effectiveness of **1**: in the case of the first stage compounds there is a significant increase in torque with respect to the reference, and in the case of final compounds, containing standard vulcanizing agents, the final torque is higher for the compound with **1**.

Figure 5c and Figure 6c show that in the first stage and final compounds, after a heating cycle at 190 °C, the addition of **1** led to a marked decrease of the Payne effect. The decrease in the Payne effect in the vulcanized compound is unexpected, given that the crosslinks between the polymer chains should increase the modulus both at low and high deformations. It was hypothesized that the decrease in the Payne effect is linked to the homogeneity of the network formed in the presence of **1**, which could reduce the possibility of interaction of the filler with itself. This decrease in the Payne effect is of technological interest as it is often associated with less hysteresis and as an indicator of lower non-linearity of the mechanical response of the tire, which will result in being more predictable, more precise, and therefore safer.

In the first stage and final compounds, after a heating cycle at the activation temperature of **1** (Figure 5d and Figure 6d), a significant reduction of the hysteresis was observed, at least at deformations greater than about 3%, which are of technological interest. The hysteresis at 70 °C is considered a predictor of the rolling resistance of the tire, making this effect newsworthy.

#### 3.4.2. Separated Temperature Curing

**Figure 7:**
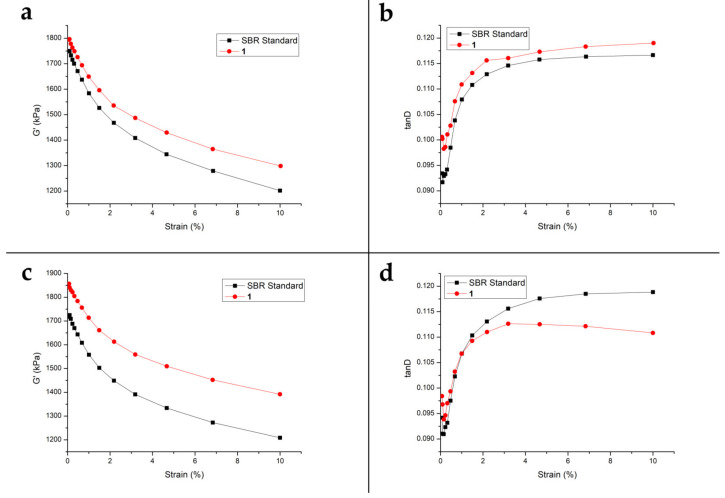
Dynamic mechanical properties of final compound treated with bistetrazole **1**: (**a**) G’ measured after curing at 150 °C; (**b**) tan delta after curing at 150 °C; (**c**) G’ measured after second curing at 190 °C; (**d**) tan delta after second curing at 190 °C.

Finally, we did a separated temperature curing on final compound with **1**, i.e., first heating at 150 °C to induce sulfur crosslinking, then heating at 190 °C to activate bis-tetrazole **1**, to evaluate the dynamic mechanical properties at the two phases (Figure 7).

After the first cross-linking at 150 °C, the hystereses of the two compounds (Figure 7a) are very similar, but the compound with **1** tends to be a little more hysteretic, as expected for the addition of a smaller molecule, which, if it remains unreacted, it should behave like a filler as seen before. Whereas the dynamic module G’ of the compounds are relatively similar (Figure 7b).

After the second cross-linking at 190 °C, the hysteresis of the compound with **1** is much lower than that of the reference standard SBR (Figure 7c). The dynamic modulus (G’) of the compound with **1** is much higher than the reference compound. While a sharp decrease in the Payne effect for the compound with **1** (Figure 7d) was observed.

## 4. Conclusions

The prepared bistetrazoles, both used as single cross-linker and together with classic sulfur-based curing systems, can significantly reduce the hysteresis of the elastomeric compounds with potential advantages in terms of lower rolling resistance of the tire. Furthermore, those bis-tetrazoles are able to significantly reduce the Payne effect of the vulcanized compounds, with potential advantages on driving precision and therefore on the safety of the tire. In fact, the cross-linking conducted with the present cross-linkers appears much more controllable than the classical sulfur cross-linking, allowing to prolong the mixing until optimum dispersion and homogeneity are obtained. It has been also demonstrated the possibility of sequential cross-linking at different temperatures, allowing in theory the production of elastomeric compounds cured with sulfur, but able to exploit a further curing at higher temperatures owing to the presence of unreacted bistetrazoles. This could translate into a smart tire where cross-link occur due to overheats in stress conditions, lowering the degrade and therefore the worsening in drive performance. Moreover, the synthetic strategy used for the preparation of bistetrazoles **1–5** starts from cheap and commercially available reagents which makes an easy scale-up to grams on a laboratory scale possible, and in principle scalable to kilograms at industrial level.

## 5. Patents

The research presented in this work leaded to the international patent WO2021137143A1.

## Figures and Tables

**Table 1:** Detected activation temperatures of **1–5**.

Compound	Activation Temperature (T_a_)
**1**	185 °C
**2**	185 °C
**3**	170 °C
**4**	190 °C
**5**	230 °C

**Table 2:** Components of first stage elastomeric compounds.

Elastomer	SSBR ^1^	Silica ^1^	Tetrazole ^1^	TESPD ^1^
SBR standard	137	60	-	4.8
SBR + **1**	137	60	4.2	4.8
SBR + **2**	137	60	5.1	4.8
SBR + **3**	137	60	4.3	4.8
SBR + **4**	137	60	5.2	4.8

^1^ Quantities are expressed in phr.

**Table 3:** Components of final elastomeric compounds.

Elastomer	SSBR ^1^	Silica ^1^	Tetrazole ^1^	6PPD ^1^	TESPD ^1^	Stearic Acid ^1^	ZnO ^1^	CBS ^1^	Sulfur ^1^
SBR standard	137	60	-	2.5	4.8	1	2	3	1
SBR + **1**	137	60	4.2	2.5	4.8	1	2	3	1
SBR + **2**	137	60	5.1	2.5	4.8	1	2	3	1
SBR + **3**	137	60	4.3	2.5	4.8	1	2	3	1
SBR + **4**	137	60	5.2	2.5	4.8	1	2	3	1

^1^ Quantities are expressed in phr.

## Data Availability

Data available on request.

## Supplementary Materials

The following supporting information can be downloaded at: https://www.mdpi.com/article/10.3390/polym14142919/s1. Synthetic procedures for the preparation of bistetrazoles **1–5**. ^1^H NMR, ^13^C NMR, and ATR-FTIR spectra of **1–5**.
