# Supplementary material for: Thermally Activable Bistetrazoles for Elastomers Crosslinking
Source: Polymers (Basel). 2022 Jul 19;14(14):2919. doi: 10.3390/polym14142919 (PMC9323196; doi:10.3390/polym14142919)
Supplement: Supplementary file 1 [file polymers-14-02919-s001.zip › polymers-1801515-supplementary.pdf]

Supplementary Materials

Thermally Activable Bistetrazoles for Elastomers Crosslinking

Mauro Monti [1], Luca Giannini [2], Luciano Tadiello [2], Silvia Guerra [2], Antonio Papagni [1] and Luca Vaghi [1,*]

[1] Department of Materials Science, University of Milano-Bicocca, Via Cozzi 55, 20125 Milano; mauro.monti@unimib.it (M.M.); antonio.papagni@unimib.it (A.P.)
[2] Pirelli Tyre S.p.A., 25-20126 Milano, Italy; Luca.Giannini@pirelli.com (L.G.); luciano.tadiello@pirelli.com (L.T.); silvia.guerra@pirelli.com (S.G.)

Table of Contents

S2-S3. Procedures for the synthesis of 1-5.

S4-S11. ^{1}H NMR, ^{13}C NMR and ATR-FTIR spectra of 1-5.

Synthetic procedures

General information

All reagents and solvents for synthesis were purchased from commercial sources (Merck Life Science S.r.l., Milan, Italy; Fluorochem Ltd., Hadfield, UK; and TCI Europe N.V., Zwijndrecht, Belgium) and used without further purification. Chromatographic purifications were performed using Merck 9385 silica gel, pore size 60 Å (230–400 mesh) (Merck Life Science S.r.l., Milan, Italy). Melting points were measured with a Stanford Research Systems Optimelt apparatus (SRS, Sunnyvale, CA, USA). IR spectra were recorded with a PerkinElmer Spectrum 100 FT-IR spectrometer equipped with universal ATR sampling accessory (PerkinElmer Inc., Waltham, MA, USA). ^{1}H and ^{13}C spectra were recorded with a Bruker AVANCE III HD 400 MHz spectrometer (^{1}H: 400 MHz, ^{13}C: 101 MHz) (Bruker corp., Billerica, MA, USA), chemical shifts (δ) are expressed in parts per million (ppm), and coupling constants are given in Hz. Splitting patterns are indicated as follows: s = singlet, d = doublet, t = triplet, q = quartet, m = multiplet, br = broad. Elemental analyses were obtained with an Elementar vario MICRO cube instrument (Elementar Analysensysteme GmbH, Langenselbold, Germany). TGA analyses were performed using Mettler Toledo TGA/DSC1 StarE (Mettler-Toledo International Inc., Greifensee, Switzerland).

*1,4-bis(2-phenyl-2H-tetrazol-5-yl)benzene (**1**).* A mixture of terephthalaldehyde (1.00 g, 7.5 mmol) and *p*-toluenesulfonyl hydrazide (2.92 g, 15 mmol) in EtOH (35 mL) was heated at reflux under stirring for 5 h. After cooling to rt, the mixture was poured into cold water. The precipitate formed was recovered by filtration and dried at 80 °C overnight. The precipitate was then dissolved in 30 mL of pyridine to give solution A. In parallel, a solution of $NaNO_2$ (1.03 g, 15 mmol) in water (4 mL) was added dropwise to a cooled (0 °C) mixture of aniline (1.40 g, 15 mmol), concentrated HCl (37% in H_2O, 3.75 mL, 45 mmol), H_2O (5 mL) and EtOH (5 mL) to give solution B. Solution A was cooled with an ice bath and solution B was then slowly added. The mixture was stirred overnight at rt. The mixture was then poured into an aqueous HCl solution (200 mL, 3 M) and the precipitate was recovered by filtration. Crystallization (EtOAc) afforded **1** in a pure form (1.51 g, 55%). Pink solid; mp: 184-185 °C dec; ^{1}H NMR (400 MHz, $CDCl_3$) δ 8.44 (s, 4H), 8.26 – 8.22 (m, 4H), 7.64 – 7.58 (m, 4H), 7.57 – 7.50 (m, 2H). ^{13}C NMR (101 MHz, $CDCl_3$) δ 164.6, 149.7, 136.8, 136.1, 129.8, 129.7, 127.6, 123.8, 119.9. IR (ATR): 3191, 1596, 1560, 1492, 1471, 1450, 1425, 1361, 1322, 1300, 1277, 1213, 1187, 1165, 1093, 1053, 1011, 994, 954, 913, 854, 835, 811, 759, 739, 702, 677,571, 553 cm^{-1}; Anal. Calcd. for $C_{20}H_{14}N_8$: C, 65.56; H, 3.85; N, 30.58. Found: C, 65.67; H, 3.89; N, 30.45.

*4,4'-bis(2-phenyl-2H-tetrazol-5-yl)-1,1'-biphenyl (**2**).* A mixture of (1,1'-biphenyl)-4,4'-dicarbaldehyde (2.50 g, 12 mmol) and *p*-toluenesulfonyl hydrazide (4.43 g, 24 mmol) in EtOH (80 mL) was heated at reflux under stirring for 5 h. After cooling to rt, the mixture was poured into cold water. The precipitate formed was recovered by filtration and dried at 80 °C overnight. The precipitate was then dissolved in 90 mL of pyridine to give solution A. In parallel, a solution of $NaNO_2$ (1.65 g, 24 mmol) in water (6 mL) was added dropwise to a cooled (0 °C) mixture of aniline (2.22 g, 24 mmol), concentrated HCl (37% in H_2O, 6 mL, 72 mmol), H_2O (10 mL) and EtOH (10 mL) to give solution B. Solution A was cooled with an ice bath and solution B was then slowly added. The mixture was stirred overnight at rt. The mixture was then poured into an aqueous HCl solution (400 mL, 3 M) and the precipitate was recovered by filtration. Crystallization (MeOH) afforded **2** in a pure form (2.39 g, 45%). Pink solid; mp: 186-187 °C dec; ^{1}H NMR (400 MHz, DMSO-d_6) δ 8.34 (d, *J* = 8.0 Hz, 4H), 8.21 (d, *J* = 7.6 Hz, 4H), 8.07 (d, *J* = 8.0 Hz, 4H), 7.73 (t, *J* = 7.5 Hz, 4H), 7.67 (t, *J* = 7.2 Hz, 2H); IR (ATR): 3065, 3032, 2050, 1697, 1615, 1597, 1535, 1494, 1458, 1431, 1409, 1374, 1360, 1318, 1293, 1253, 1210, 1184, 1165, 1139, 1106, 1087, 1075, 1033, 1013, 992, 909, 863, 825, 748, 731, 715, 692, 675, 575 cm^{-1}; Anal. Calcd. for $C_{26}H_{18}N_8$: C, 70.58; H, 4.10; N, 25.32. Found: C, 70.70; H, 4.07; N, 25.21.

*5,5'-thiene-2,5-diylbis(2-phenyl-2H-tetrazole) (**3**).* A mixture of thiophene-2,5-dicarbaldehyde (2.50 g, 18 mmol) and *p*-toluenesulfonyl hydrazide (6.64 g, 36 mmol) in EtOH (80 mL) was heated at reflux under stirring for 5 h. After cooling to rt, the mixture was poured into cold water. The precipitate formed was recovered by filtration and dried at 80 °C overnight. The precipitate was then dissolved in 100 mL of pyridine to give solution A. In parallel, a solution of $NaNO_2$ (2.48 g, 36 mmol) in water (8 mL) was added dropwise to a cooled (0 °C) mixture of aniline (3.32 g, 36 mmol), concentrated HCl (37% in H_2O, 9 mL, 108 mmol), H_2O (10 mL) and EtOH (10 mL) to give solution B. Solution A was cooled with an ice bath and solution B was then slowly added. The mixture was stirred overnight at rt. The mixture was then poured into an aqueous HCl solution (400 mL, 3 M) and the

precipitate was recovered by filtration. Crystallization (EtOAc) afforded **3** in a pure form (3.15 g, 47%). Yellow solid; mp: 170-171 °C dec; ^{1}H NMR (400 MHz, DMSO- d_6) δ 8.24 – 8.13 (m, 4H), 8.07 (s, 2H), 7.79 – 7.69 (m, 4H), 7.69 – 7.61 (m, 2H); ^{13}C NMR (101 MHz, DMSO- d_6) δ 160.4, 136.4, 131.0, 130.8, 130.7, 130.2, 120.6; IR (ATR): 3085, 2891, 1590, 1576, 1481, 1458, 1422, 1380, 1363, 1335, 1304, 1266, 1240, 1226, 1187, 1167, 1162, 1123, 1095, 1068, 1008, 967, 935, 926, 912, 815, 752, 745, 702, 673, 589, 575, 554; Anal. Calcd. for $C_{18}H_{12}N_8S$: C, 58.05; H, 3.25; N, 30.09. Found: C, 58.22; H, 3.24; N, 29.99.

*5,5'-(2,2'-bithiene-5,5'-diyl)bis(2-phenyl-2H-tetrazole) (**4**).* A mixture of [2,2'-bithiophene]-5,5'-dicarbaldehyde (2.50 g, 11 mmol) and *p*-toluenesulfonyl hydrazide (4.19 g, 22 mmol) in EtOH (90 mL) was heated at reflux under stirring for 5 h. After cooling to rt, the mixture was poured into cold water. The precipitate formed was recovered by filtration and dried at 80 °C overnight. The precipitate was then dissolved in 100 mL of pyridine to give solution A. In parallel, a solution of $NaNO_2$ (1.55 g, 22 mmol) in water (6 mL) was added dropwise to a cooled (0 °C) mixture of aniline (2.10 g, 22 mmol), concentrated HCl (37% in H_2O, 6 mL, 72 mmol), H_2O (10 mL) and EtOH (10 mL) to give solution B. Solution A was cooled with an ice bath and solution B was then slowly added. The mixture was stirred overnight at rt. The mixture was then poured into an aqueous HCl solution (400 mL, 3 M) and the precipitate was recovered by filtration. Crystallization (MeOH) afforded **4** in a pure form (2.40 g, 48%). Yellow solid; mp: 189-190 °C dec; ^{1}H NMR (400 MHz, $CDCl_3$) δ 8.23 – 8.16 (m, 4H), 7.86 (d, J = 3.8 Hz, 2H), 7.64 – 7.56 (m, 4H), 7.56 – 7.49 (m, 2H), 7.36 (d, J = 3.8 Hz, 2H); IR (ATR): 3065, 3051, 1596, 1564, 1498, 1481, 1464, 1424, 1368, 1318, 1296, 1263, 1214, 1181, 1131, 1069, 1058, 1006, 910, 879, 810, 753, 743, 701, 673, 646, 589, 573; Anal. Calcd. for $C_{22}H_{14}N_8S_2$: C, 58.14; H, 3.10; N, 24.65. Found: C, 58.35; H, 3.14; N, 24.48.

*5-phenyl-2H-tetrazole (**6**).* A mixture of benzonitrile (2.50 g, 24 mmol), sodium azide (1.73 g, 24 mmol) and zinc bromide (5.46 g, 24 mmol) in H_2O (50 mL) was heated at reflux under stirring for 24 h. The mixture was then cooled with an ice bath and acidified with concentrated HCl (37% H_2O) until pH ≈ 1. The mixture was extracted with EtOAc (3X 20 mL), the organic layer was dried (Na_2SO_4), and the solvent eliminated under reduced pressure. The crude was dissolved aqueous NaOH (200 mL, 0.25 M). The zinc salts formed were filtered off and the aqueous phase was acidified with aqueous HCl (400 mL, 3 M). The precipitate was recovered by filtration and dried at 80°C overnight to afford **6** in a pure form (2.64 g, 76 %). White solid; ^{1}H NMR (400 MHz, DMSO-d6) δ 8.08 – 8.04 (m, 2H), 7.64 – 7.57 (m, 3H); IR (ATR): 3130, 3056, 2980, 2905, 2834, 2794, 2763, 2684, 2649, 2601, 2543, 2480, 2450, 1898, 1857, 1824, 1765, 1713, 1609, 1563, 1485, 1466, 1439, 1409, 1288, 1256, 1084, 1055, 1035, 1015, 989, 956, 925, 840, 790, 784, 725, 703, 685 cm^{-1}. The physical and spectroscopic data corresponded to those reported in the literature [25].

*1,6-bis(5-phenyl-2H-tetrazol-2-yl)hexane (**5**).* 5-phenyl-2*H*-tetrazole **6** (0.76 g, 5.2 mmol) was dissolved in dry DMF (15 mL), under nitrogen atmosphere. K_2CO_3 (0.79 g, 5.7 mmol) was added in one portion and 1,6-dibromohexane (0.40 mL, 2.6 mmol) was added dropwise over a period of 10 min. The mixture was stirred at rt for 48 h. The mixture was then extracted with EtOAc (2X 20 mL), the organic layer was washed with brine (2X 20 mL), dried (Na_2SO_4) and the volatiles eliminated under reduced pressure. Flash column chromatography (SiO_2, CH_2Cl_2) afforded **3** in a pure form (0.51 g, 52%). White solid; mp: 210-211 °C dec; ^{1}H NMR (400 MHz, $CDCl_3$) δ 8.17 – 8.11 (m, 4H), 7.52 – 7.45 (m, 6H), 4.65 (t, *J* = 7.0 Hz, 4H), 2.13 – 2.02 (m, 4H), 1.49 – 1.42 (m, 4H); ^{13}C NMR (101 MHz, $CDCl_3$) δ 165.1, 130.3, 128.9, 127.4, 126.8, 52.9, 29.1, 25.7; IR (ATR): 3067, 3034, 2947, 2872, 2863, 2165, 1981, 1962, 1895, 1822, 1767, 1716, 1653, 1610, 1585, 1528, 1463, 1449, 1397, 1365, 1352, 1339, 1306, 1286, 1251, 1206, 1177, 1131, 1103, 1070, 1042, 1030, 1000, 997, 921, 855, 787, 759, 729, 689, 617 cm^{-1}; Anal. Calcd. for $C_{20}H_{22}N_8$: C, 64.15; H, 5.92; N, 29.93. Found: C, 64.36; H, 5.89; N, 29.82.

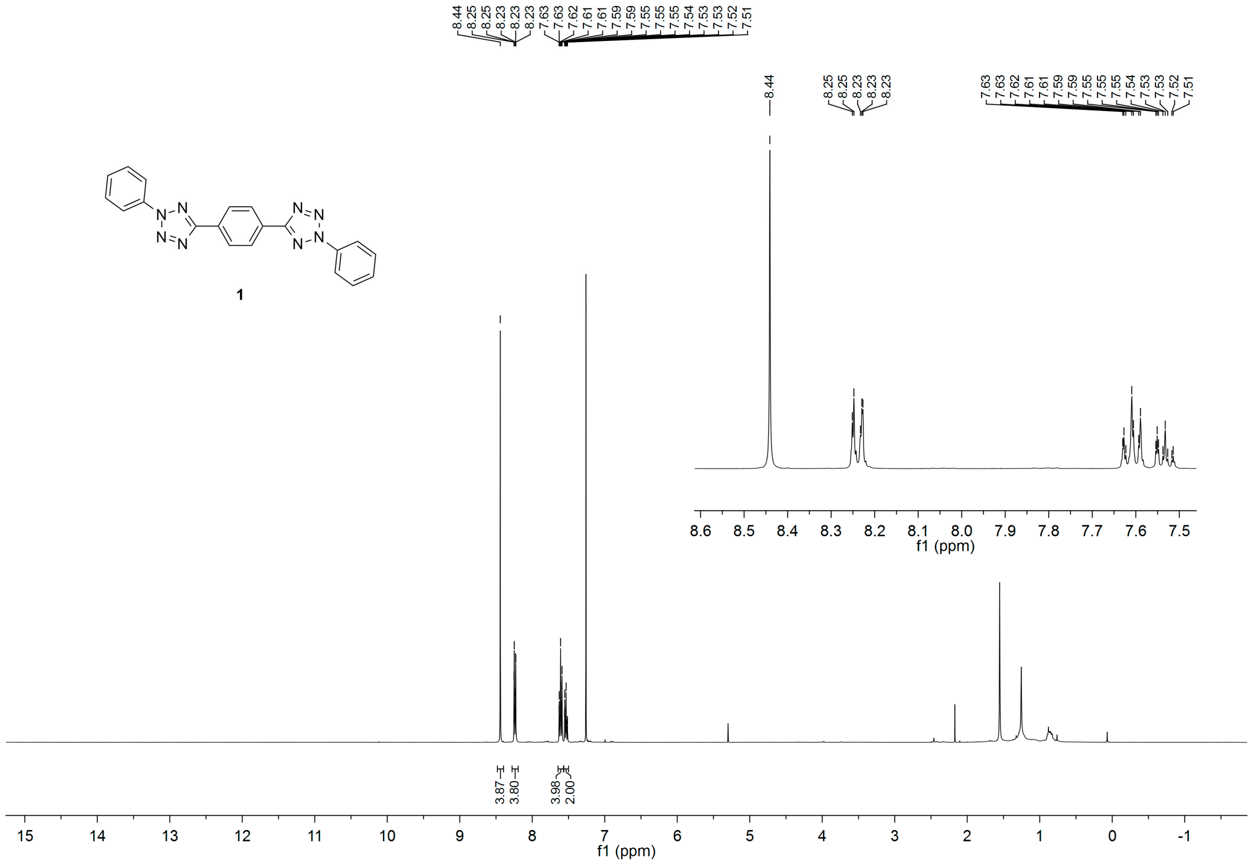

1
8.44
8.25
8.25
8.23
8.23
8.23
7.63
7.63
7.62
7.61
7.61
7.59
7.59
7.55
7.55
7.55
7.54
7.53
7.53
7.52
7.51
3.87
3.80
3.98
2.00
f1 (ppm)

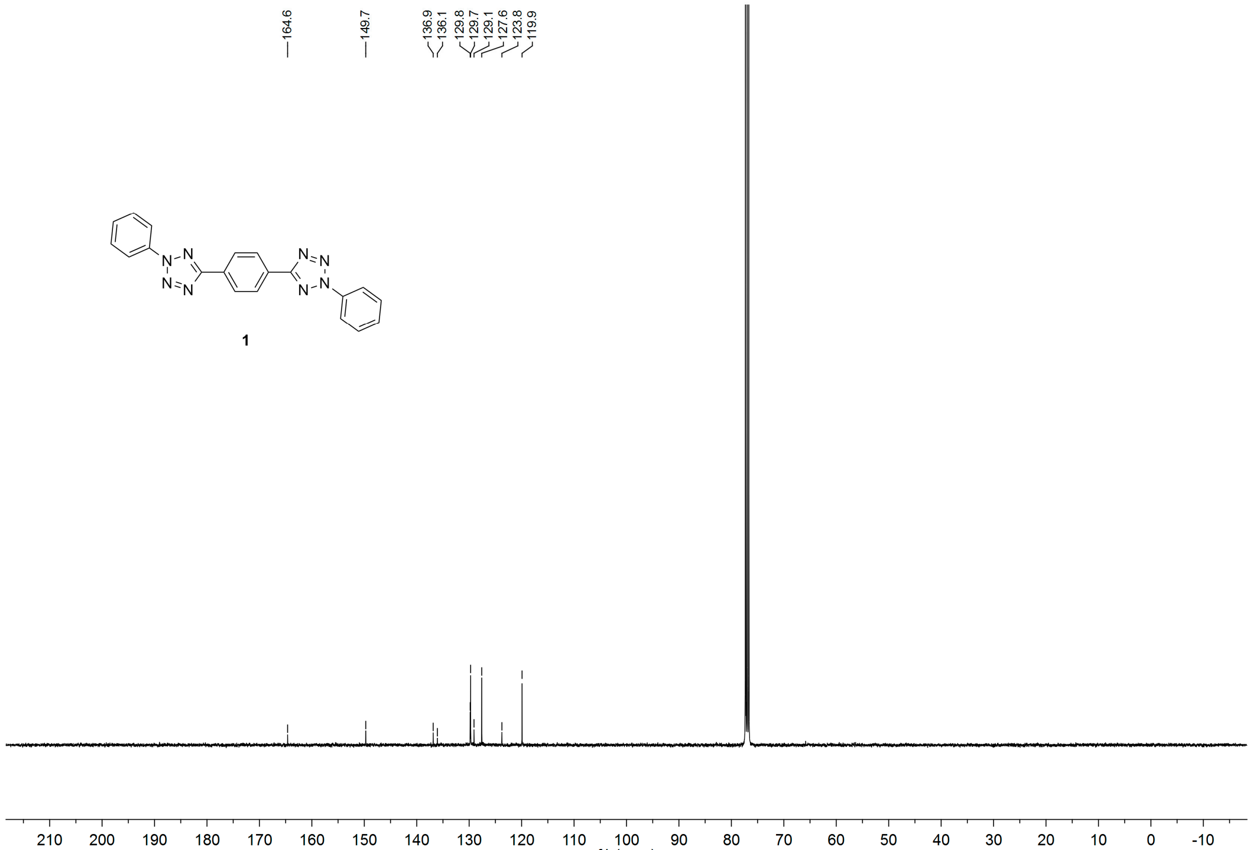

1
164.6
149.7
136.9
136.1
129.8
129.7
129.1
127.6
123.8
119.9
f1 (ppm)

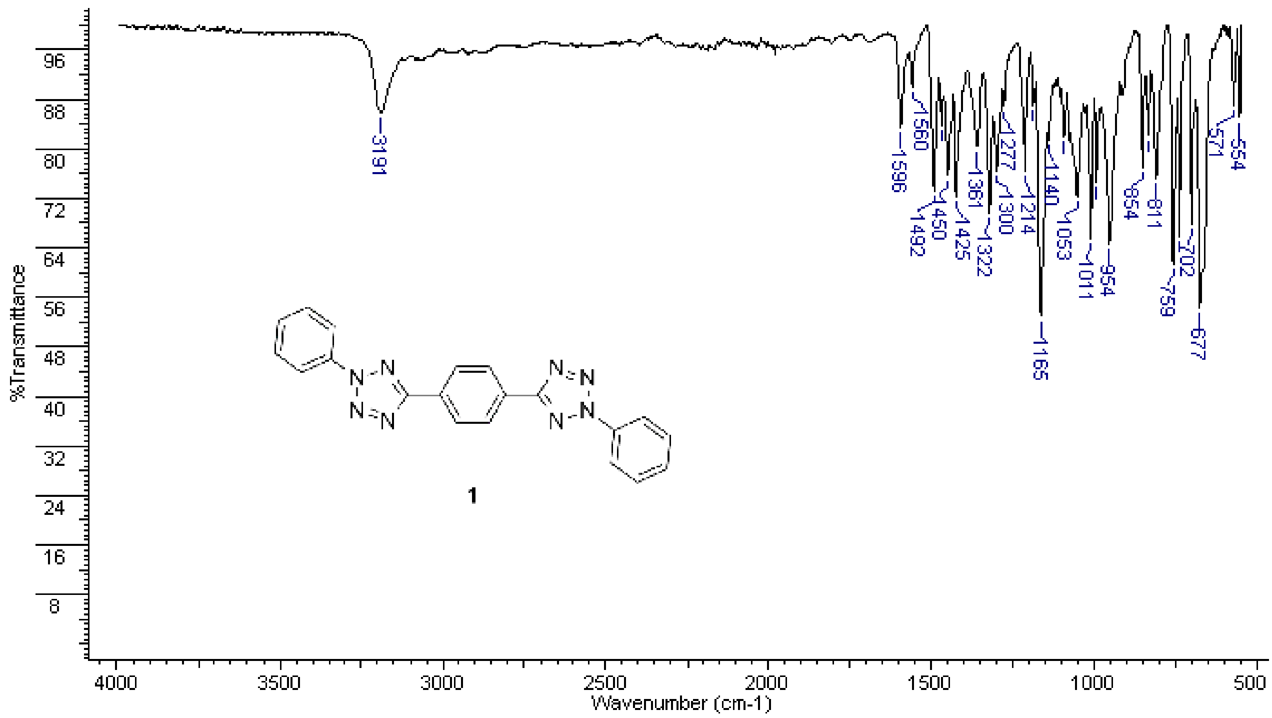
%Transmittance
Wavenumber (cm-1)
96
88
80
72
64
56
48
40
32
24
16
8
4000
3500
3000
2500
2000
1500
1000
500
3191
1596
1560
1492
1450
1425
1361
1322
1300
1277
1214
1165
1140
1053
1011
954
854
811
759
702
677
571
554
1

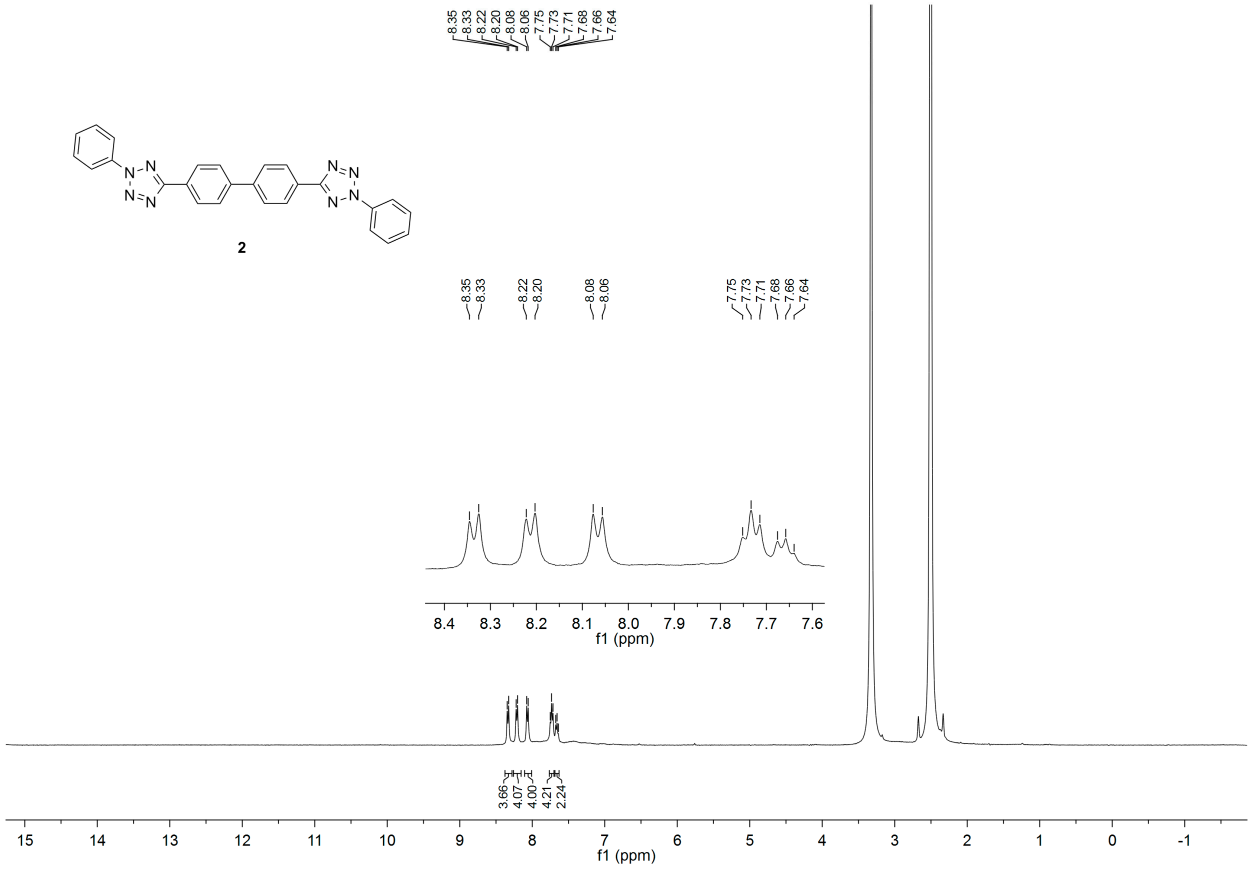
8.35
8.33
8.22
8.20
8.08
8.06
7.75
7.73
7.71
7.68
7.66
7.64
2
8.4 8.3 8.2 8.1 8.0 7.9 7.8 7.7 7.6
f1 (ppm)
3.66
4.07
4.00
4.21
2.24
15 14 13 12 11 10 9 8 7 6 5 4 3 2 1 0 -1
f1 (ppm)

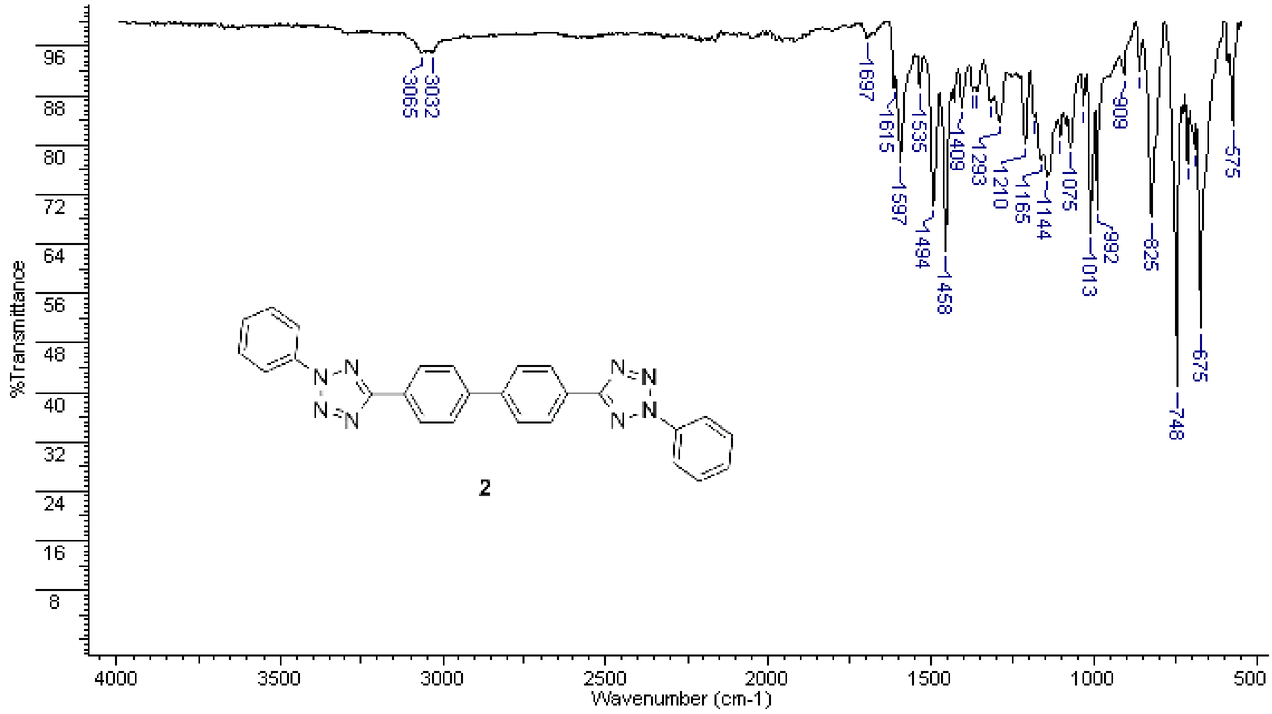
%Transmittance
Wavenumber (cm-1)
3065
3032
1697
1615
1597
1535
1494
1458
1409
1293
1210
1165
1144
1075
1013
992
909
825
748
675
575
2

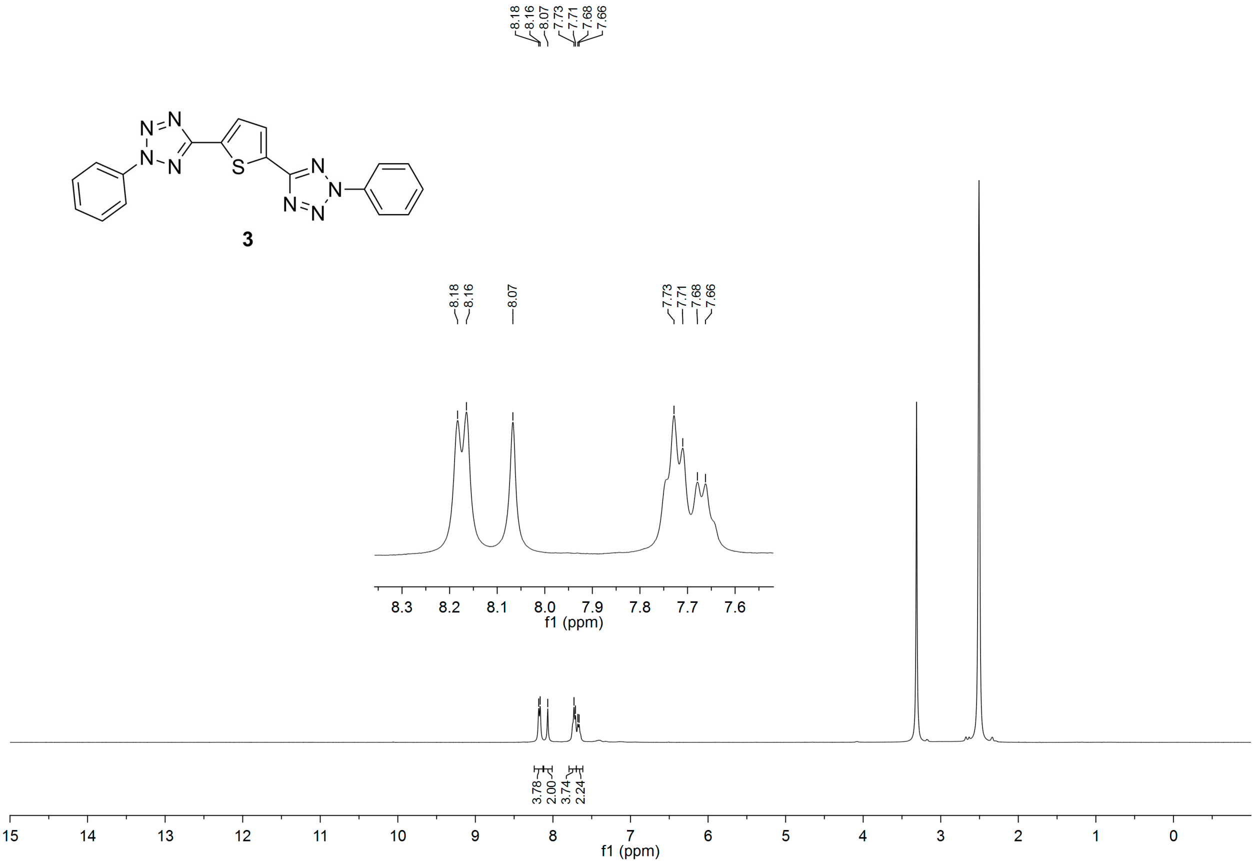
8.18
8.16
8.07
7.73
7.71
7.68
7.66
3
3.78
2.00
3.74
2.24
f1 (ppm)

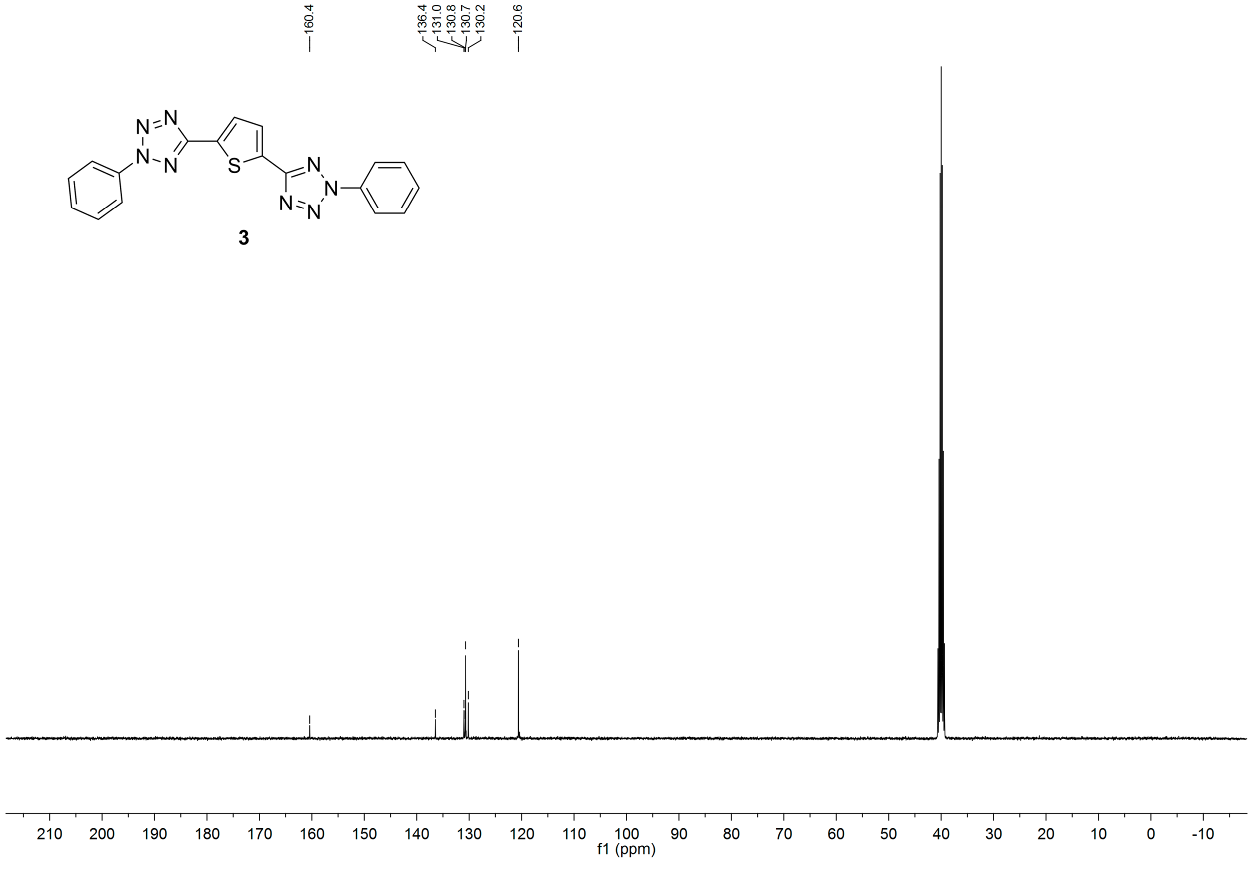
160.4
136.4
131.0
130.8
130.7
130.2
120.6
3
f1 (ppm)

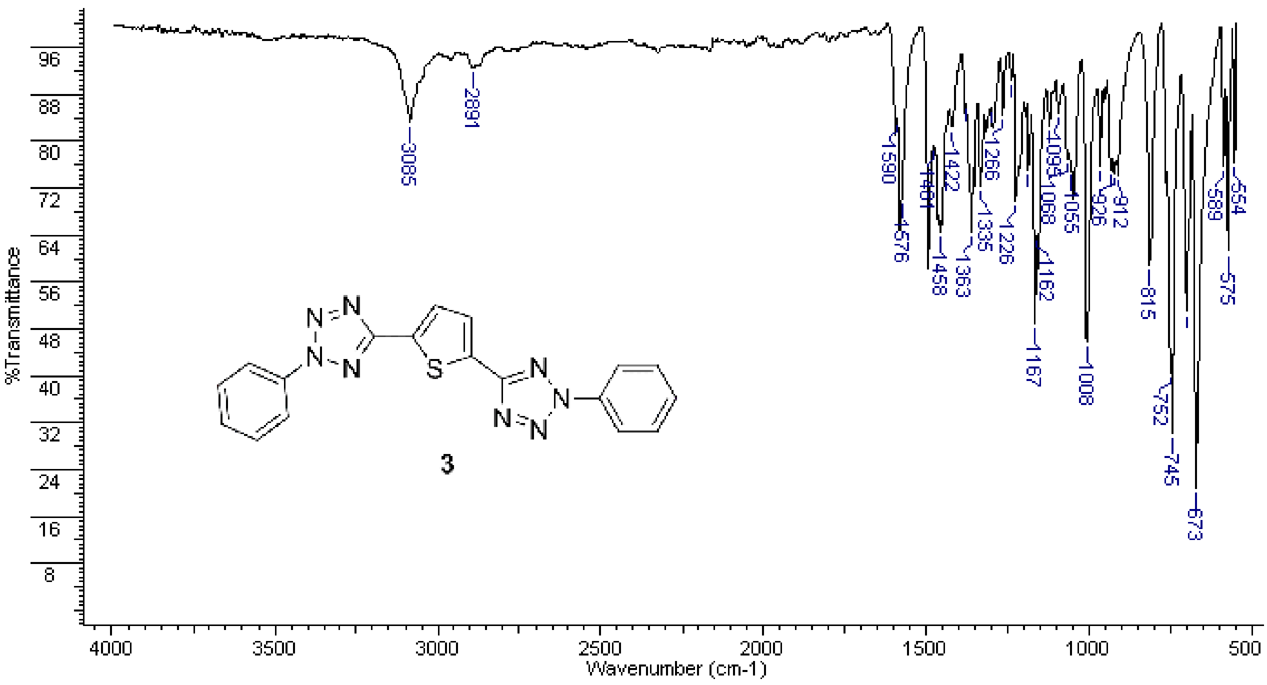

%Transmittance
96
88
80
72
64
56
48
40
32
24
16
8
4000
3500
3000
2500
2000
1500
1000
500
Wavenumber (cm-1)
3085
2891
1590
1576
1481
1458
1422
1363
1335
1266
1226
1167
1162
1093
1068
1055
1008
926
912
815
752
745
673
589
575
554
3

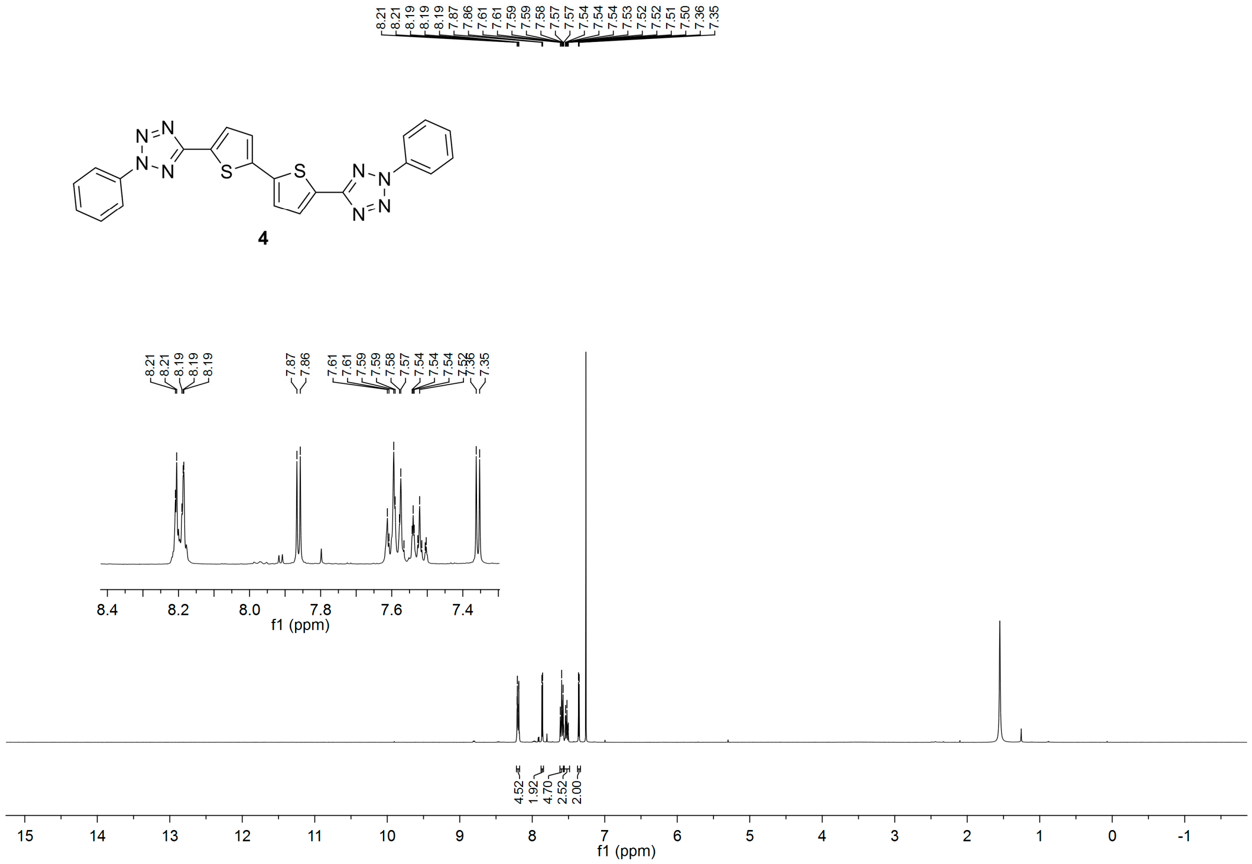
8.21
8.21
8.19
8.19
8.19
7.87
7.86
7.61
7.61
7.59
7.59
7.58
7.57
7.57
7.54
7.54
7.54
7.53
7.52
7.52
7.51
7.50
7.36
7.35
4
f1 (ppm)
4.52
1.92
4.70
2.52
2.00

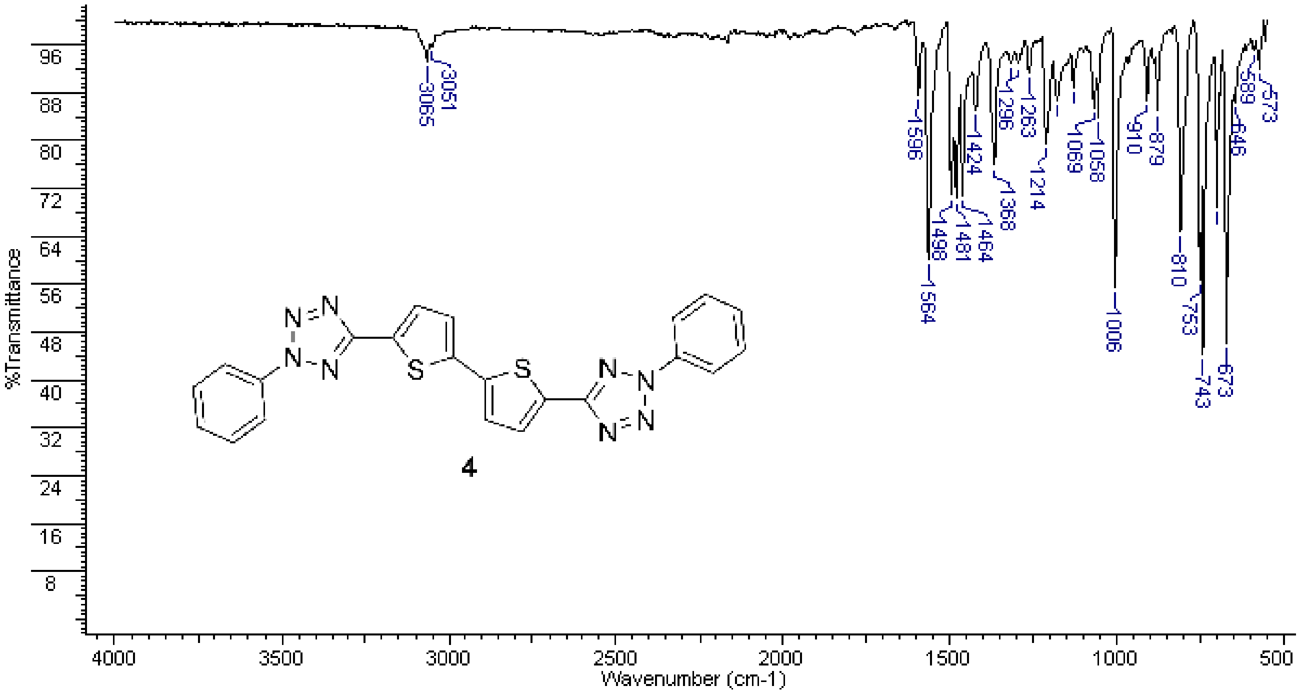
%Transmittance
Wavenumber (cm-1)
3065
3051
1596
1564
1498
1481
1464
1424
1368
1296
1263
1214
1069
1058
1006
910
879
810
753
743
673
646
589
573
4

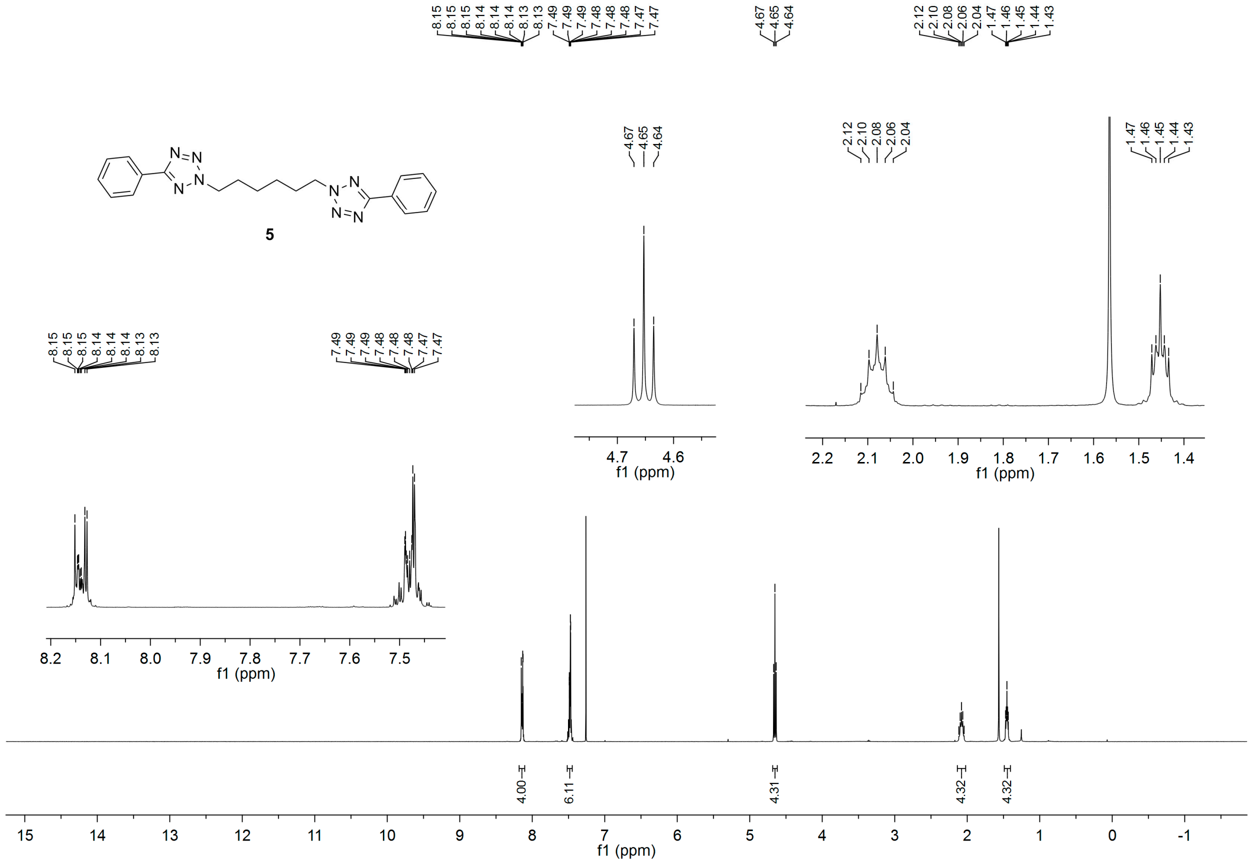
8.15 8.15 8.15 8.14 8.14 8.14 8.13 8.13 7.49 7.49 7.49 7.48 7.48 7.48 7.47 7.47
4.67 4.65 4.64
2.12 2.10 2.08 2.06 2.04 1.47 1.46 1.45 1.44 1.43
N N N N N N N N
5
4.00 6.11 4.31 4.32 4.32
f1 (ppm)

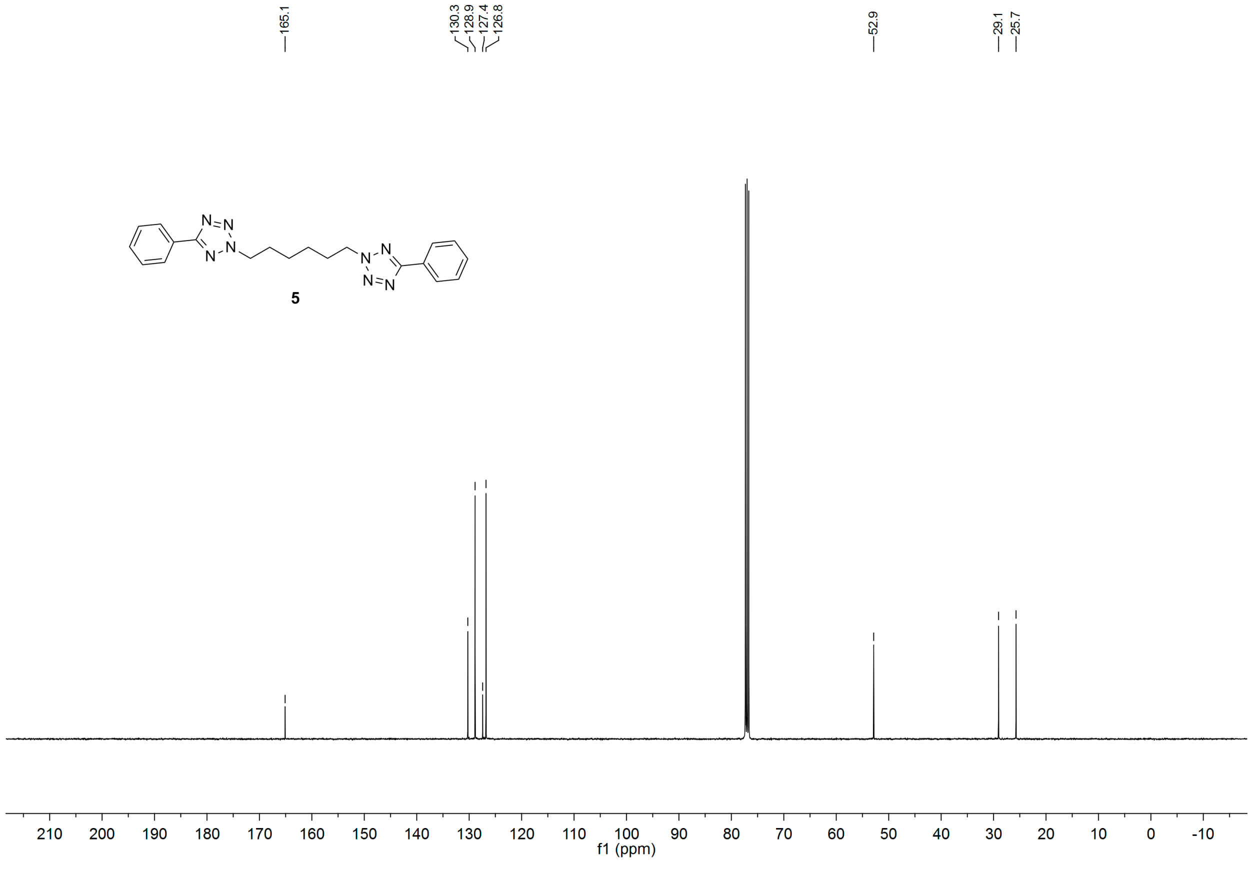
165.1
130.3 128.9 127.4 126.8
52.9
29.1 25.7
N N N N N N N N
5
f1 (ppm)

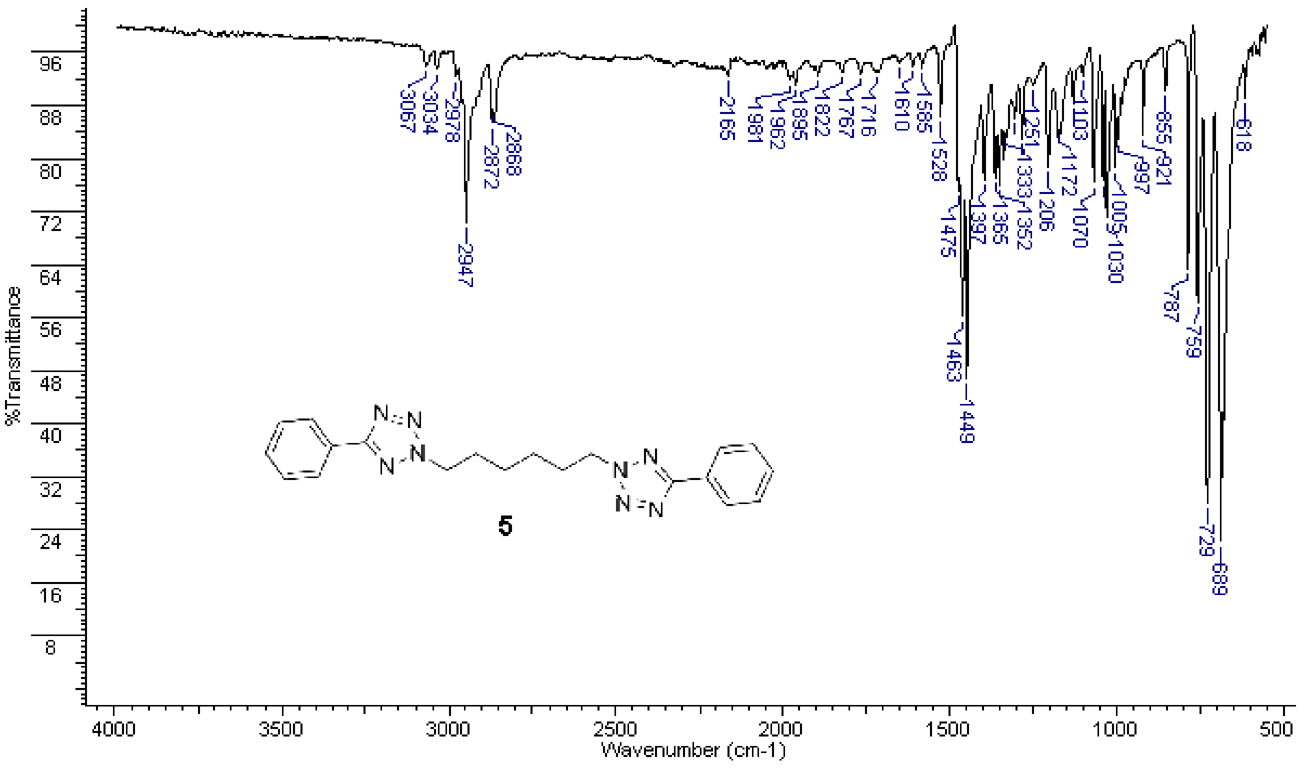
%Transmittance
Wavenumber (cm-1)
5
